# Seasonal Variations in the Foraging Strategies of Plateau Pikas (*Ochotona curzoniae*)

**DOI:** 10.3390/ani15070902

**Published:** 2025-03-21

**Authors:** Longming Dong, Xincheng Cai, Ruixun Gan, Jing Zhang, Rui Dong, Kechi Dong, Limin Hua, Rui Zhou

**Affiliations:** 1 College of Grassland Science, Gansu Agricultural University, Key Laboratory of Grassland Ecosystem of the Ministry of Education, Engineering and Technology Research Center for Alpine Rodent Pest Control of National Forestry and Grassland Administration, Lanzhou 730070, China; donglm_gsau@163.com (L.D.); caixincheng_gsau@163.com (X.C.); ganrx_gsau@163.com (R.G.); zhangj_gsau@163.com (J.Z.); dongrui_gsau@163.com (R.D.); dongkc_gsau@163.com (K.D.); 2 Academy of Animal Science and Veterinary, Qinghai University, State Key Laboratory of Plateau Ecology and Agriculture, Xining 810016, China

**Keywords:** plateau pika, stable isotope, foraging habits, trophic niche

## Abstract

This study investigates the foraging habits and dietary niche characteristics of plateau pikas (*Ochotona curzoniae*) by analyzing differences between the cold and warm seasons using stable isotope analysis of carbon and nitrogen in liver, muscle, and fur organs. The results indicated that in the cold season, the plant species incorporated into different organs were more diverse than in the warm season, with varying contribution rates for each plant species. *Potentilla anserina* made the most significant contribution to the plateau pika’s diet during the cold season, while *Kobresia humilis* had the highest contribution in the warm season. The trophic niche width of liver and muscle organs was broader in the cold season compared to the warm season, while the trophic niche width of the fur organ was narrower in the cold season. Additionally, long-term dietary information derived from muscle and fur organs showed a wider niche breadth compared to short-term dietary information obtained from the liver organ. Across both seasons, organ niche overlap was highest in fur, followed by muscle, and lowest in the liver. In conclusion, short-term food source information (derived from the liver organ) indicated fewer food types and a smaller niche breadth. In contrast, long-term food source information (obtained from muscle and fur organs) included a greater variety of food types and a larger niche breadth. Additionally, niche overlap was more pronounced on the long-term scale compared to the short-term scale. These findings highlight the adaptive feeding strategies of plateau pikas in response to varying seasonal food availability and the ecological importance of their dietary flexibility in maintaining energy balance in challenging environments.

## Simple Summary

This study uses stable isotope analysis to examine the foraging habits and dietary niche of plateau pikas (*Ochotona curzoniae*) in cold and warm seasons. In the cold season, pika diets were more diverse, with *Potentilla anserina* dominant. *Kobresia humilis* was most significant in the warm season. Liver and muscle showed broader trophic niche widths in the cold season, while fur’s was narrower. Muscle and fur (representing long-term food sources) exhibited broader niche widths than liver (short-term). Fur had the highest niche overlap, followed by muscle, then liver. These findings show pikas’ adaptive feeding strategies in response to seasonal food availability, highlighting their dietary flexibility for energy balance in harsh environments.

## 1. Introduction

Food resources are fundamental to the survival and reproduction of animals and serve as one of the key drivers in the differentiation of species’ trophic niches. The trophic niche not only reflects an animal’s role within an ecosystem, its trophic level, and the interspecies relationships, but also its specific trophic requirements [1]. For instance, rabbits (*Oryctolagus cuniculus*), deer (*Cervus elaphus*), and elephants (*Loxodonta africana*) usually play a crucial role in the formation of vegetation structure and nutrient cycling in the ecosystem. Therefore, studying the trophic niche of animals is critical for understanding their foraging strategies within the ecosystem [2]. Niche overlap and niche breadth are crucial parameters used to describe the trophic niche, as they reflect both a species’ competitive ability for food resources and the diversity of food sources exploited by animals [3]. Research on animal foraging behaviors lies at the heart of trophic niche studies and provides the foundational framework for analyzing species distribution, trophic level, and food resource availability [4]. The selection of food resources by animals is influenced by various factors, including basal metabolic rate, habitat type, predation, competition, and resource availability [5]. Thus, the study of foraging habits must not only consider short-term food selection but also address the dynamic changes in foraging strategies over the course of an animal’s life cycle, thereby determining its daily and seasonal activity [6]. This comprehensive approach offers valuable insights into the foraging strategies of species during their growth and their trophic position within the ecosystem.

Earlier studies on the trophic niche of animals utilized methods such as field observations, the microtissue method of stomach contents, laboratory cage feeding, and fecal microtissue analysis [7]. However, these techniques have limitations in reflecting the long-term foraging behavior of animals and in accurately identifying the types of excreta and stomach contents. Stable isotope analysis (SIA), which emerged in the 1970s, has become a widely employed method in animal dietary studies, trophic level assessments, and migration tracking. This method analyzes stable isotopes of δ^13^C and δ^15^N in animal organs and plants to trace animal food sources [8]. In comparison to traditional dietary study techniques, stable isotope analysis not only reveals the inter-relationships between plants and animals [9], but also provides insights into the dietary profile of individual animals over varying time scales. This is due to the different metabolic and isotopic integration rates in various organs such as liver, muscle, and fur [10].

The Qinghai–Tibetan Plateau (QTP), often referred to as the ‘Roof of the World’, is a critical ecological security barrier in China, playing a vital role in climate regulation, water conservation, and the maintenance of biodiversity [11]. Alpine meadows, the dominant vegetation type of the QTP [12], provide essential ecological services [13]. The plateau pika, a small mammal native to the alpine meadow, competes with livestock for food resources, relying on pasture for foraging. When its population increases significantly, it can lead to a reduction in grassland productivity, which is why the plateau pika is often classified as a pest [14]. However, other studies suggest that the plateau pika primarily forages on non-edible plants that are not consumed by livestock, which minimizes the impact on livestock grazing. This makes studying the pika’s diet particularly important [15]. Traditional studies have utilized microhistological analysis of stomach contents, laboratory cage feeding, and fecal microhistology [16], offering insights into the pika’s short-term dietary habits and the proportion of different food items in its diet. The foraging patterns of small mammals show significant seasonal variation, with different strategies employed in different seasons [17]. Therefore, to gain a comprehensive understanding of the diets of small mammals, studies should span different time scales.

The objective of this study is to determine the stable isotopes (δ^13^C and δ^15^N) of various organs of plateau pikas and the plants in their habitats, analyze the plant species present in different organs, and assess their contribution rates. To verify the food selection and trophic niche overlap of plateau pikas under different food resource availability conditions, thereby exploring the seasonal dynamics of their trophic niche and adaptive foraging strategies. This research will provide both theoretical and technical support for understanding the relationship between plateau pikas and the plants in grassland ecosystems, and contribute to the scientific management and control of plateau pika infestations.

## 2. Materials and Methods

### 2.1. Study Area

The Qinghai–Tibetan Plateau (QTP) is a critical ecological security barrier in China, playing a vital role in climate regulation. The study area is located in the northern Qinghai–Tibet Plateau, specifically in Maqu County in Gansu Province (N 33°59′53″, E 102°02′11″). The climate is classified as continental monsoon, influenced primarily by the southeastern monsoon and high pressure from Siberia. Winters are long and severe, while summers are short and cool [18]. Based on a survey of vegetation and topography in the study area, we selected an alpine meadow pasture at an altitude of 3250 m. The relative population density of plateau pikas is estimated at 405 active burrow entrances per hectare. The dominant plant species include *Elymus nutans*, *Poa annua*, *Stipa purpurea, Kobresia graminifolia*, *Potentilla ansrina*, and *Anemone trullifolia*, among others. Companion plant species include *Ligularia virgaurea*, *Thermopsis lanceolata*, *Taraxacum officinale*, *Ranunculus tanguticus*, *Euphorbia esula*, *Lamiophlomis rotata*, *Anemone rivularis*, and *Oxytropis ochrocephala* [19].

### 2.2. Plant Sample Collection and Pretreatment

In August 2023, within the plateau pika habitat, 10 active holes were selected for plant sampling. Plant samples were collected within a 50 cm radius of the center of each hole. The samples were placed in envelope bags, labeled with the plant species name and collection date, and then transported to the laboratory. In the laboratory, the samples were oven-dried at 65 °C for 48 h, ground in an agate mortar, and sieved through a 200-mesh sieve. The resulting powder was then stored for subsequent analysis [16].

### 2.3. Animal Sample Collection and Pretreatment

A total of twenty-four plateau pikas were captured using board traps in the study area during the cool (March–April) and warm (July–August) seasons, with twelve individuals captured in each season (Table 1). Liver, hind leg muscles, and back fur were dissected, sealed in centrifuge tubes, and stored at −20 °C. All samples were dried at 65 °C for 48 h, placed in separate envelopes. The three organs were then ground separately in an agate mortar in preparation for subsequent degreasing experiments. Fur samples were treated following the degreasing methods outlined by Guo [20], while muscle and liver samples were processed according to the protocol of Schmidt [21].

### 2.4. Stable Isotope Determination

Stable carbon and nitrogen isotope ratios were measured using a stable isotope ratio mass spectrometer (PrecisION, Deggingen, Germany) and an elemental analyzer (vario ISOTOPE cube, Langenselbold, Germany) for the pretreated samples, with an accuracy of <±0.05‰ for δ^13^C and ≤±0.05‰ for δ^15^N values. The isotope ratio (δ value) was calculated using the following formula:

δ = [(Rsample/Rstandard) − 1] × 1000‰

where δ represents the ^13^C value or ^15^N value; R is the ratio of ^13^C/^12^C or ^15^N value to the ^14^N value; Rsample is the ratio for the sample; and Rstandard is the ratio for the standard. The relative standard for δ^13^C was Vienna PeeDee Belemnite (V-PDB), and the relative standard for δ^15^N was atmospheric nitrogen. To ensure the precision and accuracy of the measurements, three standard samples were analyzed for every 10 animal organs or plant samples.

### 2.5. Calculation of Stable Isotope Trophic Discrimination Factors

Stable isotope mixing models (SIMMs) are commonly used to study the trophic niche of animals. However, the accuracy of these models is heavily influenced by the selection of trophic discriminators, which refer to the shift in stable isotope ratios that occurs when consumers reach isotopic equilibrium with their food sources. In this study, the R package SIDER (Stable Isotope Discrimination Estimation using R, Version 1.0.0.1) was used to estimate the trophic discrimination factors for different organs of plateau pikas [22].

### 2.6. Screening of Potential Food Sources for Plateau Pika

When using stable isotope mixing models to assess the contribution of food sources to animal diets, increasing the number of food sources can reduce the accuracy of model estimates. Therefore, it is crucial to objectively identify food resources to provide reliable a priori information for modeling, thus improving the accuracy of predictions made by hybrid models [23]. Initially, food resources for plateau pikas were screened based on the stable isotopes δ^13^C and δ^15^N in the food items, which were found to be lower than those in the organs of the animals [16]. This was then compared with the traditional method of investigating plateau pika diets through fecal microhistological analysis [24,25], leading to the final selection of potential food resources.

### 2.7. Estimating the Tood Contribution of the Plateau Pika

The food composition of plateau pikas was estimated using the simmr package in R, which employs Bayesian mixture modeling to quantify the contribution of different food sources. These models generate a probability density function that represents the proportion of each food source in the consumer’s dietary. The median value of the probability density function is used to estimate the most likely contribution of the food source to the animal’s diet [26].

### 2.8. Ecological Niche Width and Overlap Calculations

The Shannon–Weiner diversity index was used to calculate the width of the food ecological niche, using the following formula [27]:

$$H=\sum_{i=1}^{S}P_{i}{log}_{2}P_{i}$$

where *H* represents the ecological niche width of different organs of the plateau pika, and *P_i_* is the proportion of potential food *i* to the plateau pika’s diet. The larger the value of *H*, the wider the ecological niche of the consumer.

The ecological niche overlap between species was calculated using the Pianka overlap index [27].

$$C=\frac{\sum P_{1i}P_{2i}\ldots P_{ni}}{\sqrt{\sum P_{1i}^{2}P_{2i}^{2}\ldots P_{ni}^{2}}}$$

where *C* represents the degree of ecological niche overlap between different organs of the plateau pika, and *Pn_i_* is the contribution of potential food source *i* in different organs (*n*).

### 2.9. Statistical Analysis

Excel was used to calculate, sort, and analyze the data related to the nutritional discrimination factors, food contribution rate, and nutrient niche width and overlap degree of different organs of plateau pika in the cold and warm seasons.

## 3. Results

### 3.1. Stable Isotope Trophic Discrimination Factors of Different Organs in Plateau Pikas

The trophic discrimination factors for carbon and nitrogen stable isotopes in three organs from plateau pikas were estimated (Table 2). The carbon stable isotope trophic discrimination followed the order of fur > liver > muscle. In contrast, the nitrogen stable isotope trophic discrimination factors followed the order of liver > fur > muscle.

### 3.2. Screening of Potential Food Sources of Plateau Pika in Cold and Warm Seasons

**Figure 1:**
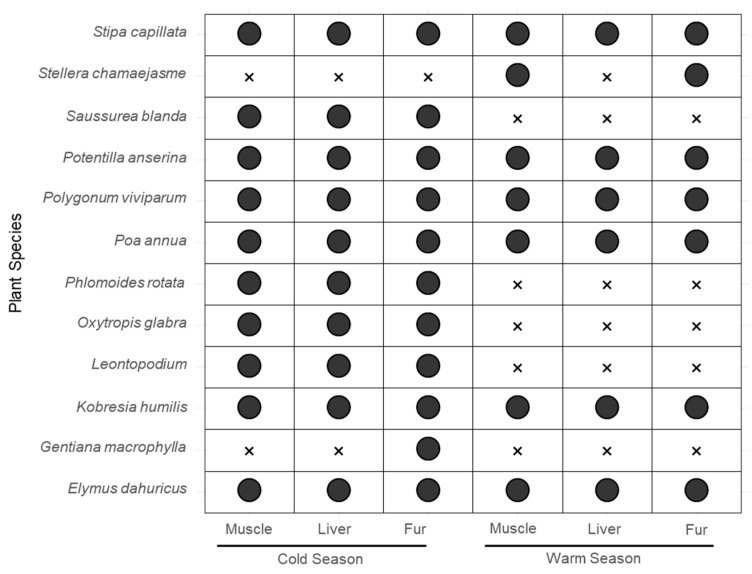
Potential food source screening of plateau pika in warm and cold seasons. Note: ● in the table represents plants foraged by plateau pikas, and ✕ represents plants not foraged.

A comparison of potential food sources in different organs of plateau pikas during the cold and warm seasons is presented in Figure 1. In the cold season, 11 plant species were identified as potential food sources, including *Elymus dahuricus*, *Stipa capillata*, and *Kobresia humilis*. In contrast, the warm season had fewer food sources, with only eight plant species, including *Leontopodium*, *Oxytropis glabra*, and *Stellera chamaejasme*. Overall, the potential food sources for plateau pikas showed minimal variation in muscle, liver, and fur organs between the cold and warm seasons.

### 3.3. Contribution of Potential Food Sources to Plateau Pikas in Cold and Warm Seasons

**Figure 2:**
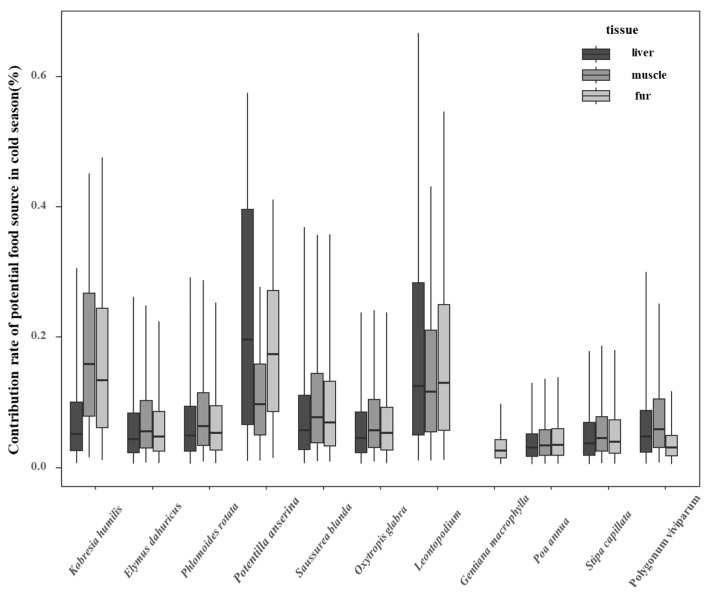
Contribution rate of potential food sources in plateau pika during the cold season.

Based on the estimated contribution rates of different food sources for plateau pikas during the cold season (Figure 2), the liver organ, reflecting short-term feeding habits, indicated the presence of seven families, 10 genera, and 10 plant species, with the following contributions: *Potentilla anserina* (23.5%), *Leontopodium* (16.7%), *Kobresia humilis* (12.1%), *Oxytropis glabra* (9.3%), *Saussurea blanda* (8.8%), *Elymus dahuricus* (8.4%), *Phlomoides rotata* (7.5%), *Polygonum viviparum* (7.0%), *Stipa capillata* (7.0%), and *Poa annua* (5.0%). Muscle organ, reflecting mid-term feeding habits, contained 10 plant species from 10 genera and seven families, including *Kobresia humilis* (11.4%), *Leontopodium* (16.5%), *Saussurea blanda* (12.5%), *Potentilla anserina* (11.9%), *Phlomoides rotata* (10.2%), *Oxytropis glabra* (9.8%), *Polygonum viviparum* (9.1%), *Elymus dahuricus* (8.9%), *Stipa capillata* (7.8%), and *Poa annua* (5.1%). The fur organ, reflecting long-term feeding habits, included 11 plant species from 11 genera and eight families, with the following contributions: *Leontopodium* (19.9%), *Potentilla anserina* (19.1%), *Kobresia humilis* (18.5%), *Saussurea blanda* (12.0%), *Oxytropis glabra* (9.4%), *Phlomoides rotata* (8.7%), *Elymus dahuricus* (7.8%), *Stipa capillata* (6.4%), *Poa annua* (5.2%), *Polygonum viviparum* (4.4%), and *Gentiana macrophylla* (3.7%). It is evident that in the short-term feeding habits, reflected by the liver and muscle organs (during the full grass and regreening periods), the main food sources include the aboveground parts of plants like *Leontopodium*, *Potentilla anserina* and *Kobresia humilis*, the proportion of the aboveground parts of *Potentilla anserina, Leontopodium*, and *Kobresia humilis* assimilated by the liver organ was 23.5%, 16.7%, and 12.1%, respectively, and decreased to 11.9%, 16.5%, and 11.4% in muscle. In contrast, the medium-term and long-term feeding habits, represented by the fur organ (during the full grass, regreening, and grass withering periods), predominantly include the aboveground parts of plants like *Leontopodium* and *Potentilla anserina*.

**Figure 3:**
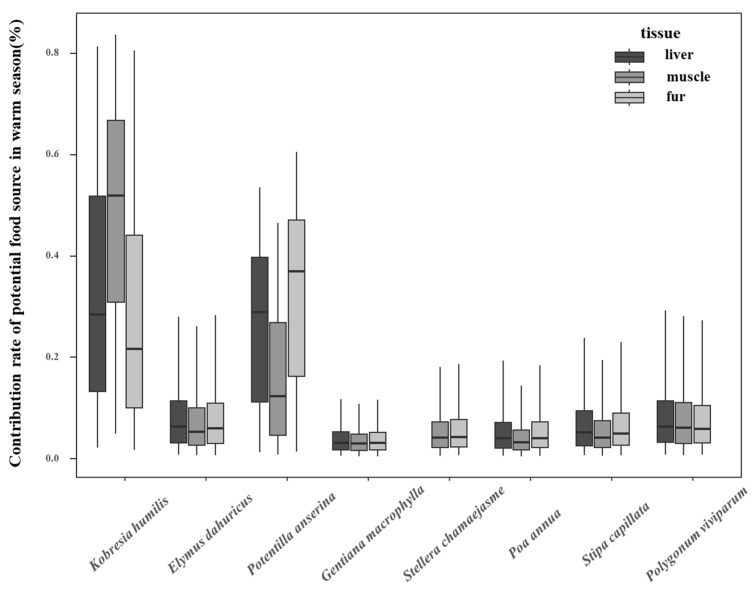
Contribution rate of potential food sources in plateau pika during warm season.

An analysis of the estimated contribution rates of different food sources during the warm season is shown in Figure 3. The liver organ, reflecting short-term feeding habits, included five families, seven genera, and seven plant species. The top three plant species in terms of contribution rates were *Kobresia humilis* (35.4%), *Potentilla anserina* (26.9%), and *Polygonum viviparum* (10.2%). The muscle organs, reflecting mid-term feeding habits, contained six families, eight genera, and eight plant species; the top three plant species in terms of contribution rates are *Kobresia humilis* (47.7%), *Potentilla anserina* (18.2%), *and Polygonum viviparum* (9.8%). The fur organ, reflecting long-term feeding habits, included eight species from eight genera and six families; the top three plant species in terms of contribution rates are *Potentilla anserina* (32.5%), *Kobresia humilis* (31.7%), *and Elymus dahuricus* (9.8%). It is clear that *Kobresia humilis*, *Potentilla anserina*, and *Polygonum viviparum* were the primary food sources across all organs of the plateau pika. In short-term feeding, as reflected by the liver organ, the proportion of *Kobresia humilis* and *Potentilla anserina* was 35.4% and 26.9%, respectively. In muscle organs, the proportion of these food sources decreased overall. However, in long-term feeding habits, as reflected by the fur organ (during the peak plant growing, regreening, and withering periods), the assimilation ratio of these food sources showed an overall increasing trend.

### 3.4. Niche Width and Overlap of Different Organs of Plateau Pika in Cold and Warm Seasons

Table 3 presents the nutrient niche breadth and overlap among muscle, liver, and fur organs of plateau pikas across different seasons. In the cold season, the niche breadth increased gradually from liver (2.585) to muscle (2.879) and fur (3.246). In the warm season, the order of increase was as follows: liver (2.032), muscle (2.034), and fur (3.478). For the food assimilated by the fur organ, the niche width of potential food sources was larger in the warm season than in the cold season. Conversely, for the liver and muscle organs, the niche width of potential food sources was greater in the cold season than in the warm season.

In the cold season, muscle–fur (0.951) and liver–fur (0.952) overlaps are highest, indicating strong dietary similarity. In the warm season, liver–fur overlap was highest (0.971), with liver–muscle overlap at 0.852. Fur consistently shows high overlap values in both seasons, reflecting stable dietary patterns, while muscle exhibits lower overlap, suggesting greater dietary divergence.

## 4. Discussion

### 4.1. Determination of Stable Isotope Nutrition Discrimination Factors in Various Organs of Plateau Pika

Trophic discrimination factors are essential for accurately determining the composition of animal diets. When properly accounted for, stable isotope data can be incorporated into analytical models to improve the precision of food source estimations [28]. Numerous studies have explored methods for obtaining these discrimination factors, with controlled feeding experiments in laboratory settings considered the most reliable approach. However, under natural conditions, the dietary intake of animals may not be accurately reflected by values derived from controlled experiments. In the absence of detailed individual data, researchers typically estimate these factors using average values from similar species or predators with comparable physiological systems [29]. Some studies have compiled trophic enrichment factors from approximately 300 individuals across 66 species to facilitate the estimation of trophic discrimination factors. However, such methods provide only generalized average values for some individuals [30]. Healy introduced a phylogenetic regression model based on compiled datasets using the R programming language to calculate trophic discrimination factors for various organs in consumers [22]. Bayesian models were then applied to determine the mean and variance of discrimination factor values across different organs. This study builds upon previous research, incorporating Δ^15^N and Δ^13^C values from studies such as Wang Zhipeng [16], Wang Xin [31], and Robb [32], to refine the estimation of trophic discrimination factors for plateau pikas.

The degree of stable isotope enrichment varies across animal organs, primarily due to differences in metabolic activity [33]. Active organs, such as the liver and muscle, typically exhibit lower enrichment levels compared to inert organs like fur. In this study, the liver and muscle organs of plateau pikas, which have higher metabolic activity, showed lower enrichment of heavy isotopes, particularly carbon isotopes. In contrast, the fur organ, which grows more slowly and does not exchange materials with the external environment once fully developed, exhibited higher enrichment levels, indicating a higher nutritional discriminant factor [20]. A similar pattern was observed for nitrogen isotopes [16].

### 4.2. Changes in Feeding Habits of Plateau Pika with Time Scale

Feeding characteristics of animals at different times are reflected in various organs. Stable isotope values in blood, liver, muscle, and fur organs can provide insights into feeding habits over different time scales, ranging from hours or days to several months [31]. In this study, the collection times of plateau pika organ samples were aligned with the corresponding feeding periods: the liver organ reflects feeding habits in August, the muscle organ captures feeding habits during the peak grass growth in August, and the fur organ represents feeding from regreening to the end of August. During these periods, plant biomass in the plateau pika habitat varied. In the study area, plant biomass shifted from reproductive to vegetative growth in August, leading to an increase in nutrient content and a reduction in the number of plant species consumed during the warm season. From regreening to the end of the grass period, plant biomass and nutrient levels decreased, prompting plateau pikas to consume more low-quality plants to meet their energy needs.

The short-term and long-term food sources of plateau pikas were dominated by plants such as *Potentilla anserina* and *Polygonum viviparum*, consistent with the tendency of small rodents to feed on plants with higher nutritional value [34]. Long-term food sources also included grasses such as Elymus dahuricus and Kobresia humilis, as plateau pikas rely on high-cellulose plants from the Gramineae and Cyperaceae families, supported by their developed cecum for digesting such food [35]. The species and contribution rates of assimilated plants varied across organs in both the cold and warm seasons. Forbs like *Kobresia humilis* and *Potentilla anserina* were dominant in both seasons. Notably, the liver organ reflected fewer plant species compared to the muscle and fur organs. During the cold season (March–April), a period of plant regreening, plant biomass was low, prompting plateau pikas to diversify their diet, which was reflected in the fur organ with 11 species. The muscle organ, representing a longer feeding period, incorporated more long-term feeding habits (10 species), while the liver organ, representing short-term feeding, reflected fewer species (7 species) [36]. In contrast, the warm season (July–August), which corresponds to the grassy period, saw an increase in plant biomass. The liver organ, reflecting short-term feeding habits at the end of August, recorded only seven plant species, whereas the fur organ, representing long-term feeding, reflected a broader range of eight plant species. This finding differs from the study of Renagül Exmet et al. [17], which showed that the number of plant species consumed by plateau pikas in the cold season was smaller than in the warm season. This discrepancy may be attributed to the prolonged livestock grazing in the cold season, which limited food availability and forced plateau pikas to expand their feeding range. Chen Guo Kang’s research on four rodent species in the Alxa Desert region revealed that the availability of food resources in the environment changes with the seasons, thereby influencing the dietary habits and seasonal variations in the trophic niches of desert rodents [37].

### 4.3. Trophic Niche Characteristics of Plateau Pika at Different Time Scales

Niche breadth and niche overlap are key indicators for describing trophic niches [38]. The results of this study indicated that the trophic niche breadth was largest for long-term dietary information, as reflected by the fur organ, followed by the muscle organ, with the smallest niche breadth found in the liver organ, which represents short-term dietary habits. In plateau zokors, the stable isotopes ^15^N and ^13^C from food sources show shorter enrichment periods in muscles and longer enrichment periods in fur. This pattern aligns with the findings from Wang Zhipeng’s study on plateau zokors [16].

In both cold and warm seasons, liver and muscle organs showed a wider niche breadth in the cold season than in the warm season. Conversely, the fur organ had a smaller niche breadth in the cold season and a larger one in the warm season. This suggests that, in the short-term diet represented by the liver organ, plateau pikas had a higher demand for plants in the cold season compared to the warm season, while in the long-term diet, as represented by the fur organ, plant demand was higher in the warm season. This observation is consistent with the timing of plateau pika and plant sample collection. At the end of August, food sources reflected by the liver organ showed a higher degree of selectivity, with *Potentilla anserina* contributing 28.9%, while selectivity was lower in the cold season. Over the long term, plateau pika diets followed a generalist feeding strategy, as the low trophic content of grassland plants during the regreening period forced plateau pikas to consume a broader range of suboptimal plants to meet their energy needs.

Studies have shown that in areas with limited food resources, small mammals tend to consume less preferred foods to avoid intraspecific competition [39]. This study found that the niche overlap of short-term food sources (represented by the liver organ) was lower in both cold and warm seasons compared to the overlap in long-term food sources (muscle and fur organs). This suggests that when food resources are abundant, plateau pikas focus on more suitable food sources, leading to different types and contributions of food resources in different seasons. In contrast, when food resources are scarce, plateau pikas increase their reliance on suboptimal foods, resulting in greater overlap in trophic niches over the long term.

## 5. Conclusions

In conclusion, plateau pikas display distinct feeding behaviors across different seasons, primarily consuming *Potentilla anserina*, *Leontopodium*, and *Kobresia humilis* in the cold season, and *Kobresia humilis*, *Potentilla anserina*, and *Polygonum viviparum* in the warm season. The seasonal foraging behavior of plateau pikas not only influences the composition of plant communities and nutrient cycling but also plays a crucial role in the food web. The analysis of short-term and long-term dietary patterns reveals significant differences in the species composition and contribution rates of ingested plants. Short-term food sources, reflected in the liver organ, involve fewer plant species and exhibit a smaller trophic niche breadth. In contrast, long-term food sources, represented by the muscle and fur organs, encompass a greater variety of species and exhibit a broader trophic niche breadth. Furthermore, niche overlap for short-term food sources is lower than for long-term sources in both the cold and warm seasons. These findings suggest that plateau pikas adapt their feeding strategies based on food availability, consuming a broader range of plants during times of resource scarcity and focusing on more selective feeding when food resources are abundant. The adaptive changes in the foraging strategies of plateau pikas reflect the dynamic balance of the ecosystem, while also providing important insights for ecological conservation and grassland management.

## Figures and Tables

**Table 1:** Individual information of plateau pika in cold and warm seasons.

Item	Cold Season		Warm Season	
	Female (*n* = 6)	Male (*n* = 6)	Female (*n* = 5)	Male (*n* = 7)
Body Mass (g)	132.1 ± 6.2	143.9 ± 13.8	139.0 ± 14.6	148.0 ± 13.1
Body Length (cm)	17.4 ± 0.3	18.1 ± 0.7	15.4 ± 1.0	17.0 ± 0.9
Hind Foot Length (cm)	2.7 ± 0.5	2.9 ± 0.5	2.7 ± 0.3	3.1 ± 0.3

**Table 2:** Distinguishing factor of stable isotope nutrition of plateau pika.

Organ	Δδ^13^C	SD^13^C	Δδ^15^N	SD^15^N
Muscle	2.126	0.862	3.354	0.522
Liver	2.148	0.865	3.428	0.526
Fur	2.957	0.911	3.391	0.504

**Table 3:** The width and overlap of nutrient niche of different organs of plateau pika in the cold and warm seasons.

Organs of Plants and Animals		Niche Breadth	Cold Season			Warm Season		
			Muscle	Liver	Fur	Muscle	Liver	Fur
Cold season	Muscle	2.879	1					
	Liver	2.585	0.844	1				
	Fur	3.246	0.951	0.952	1			
Warm season	Muscle	2.034	0.718	0.411	0.635	1		
	Liver	2.032	0.758	0.716	0.803	0.852	1	
	Fur	3.478	0.684	0.782	0.802	0.707	0.971	1

## Data Availability

The datasets used and/or analyzed during the current study are available from the corresponding author on reasonable request.
